# Patient engagement – the PaCER model

**DOI:** 10.1186/1745-6215-16-S3-O2

**Published:** 2015-11-24

**Authors:** Chris Hylton

**Affiliations:** 1 PaCER, O'Brien Institute for Public Health, University of Calgary, Calgary, Canada

## Background

Training citizens living with various health conditions to design and conduct health research, using adapted methods of qualitative inquiry, paves the way for new approaches. Patient and Community Engagement Researchers (PaCERs) collaborate with health professionals, policy makers and engage other patients in all phases of research from setting study questions, agendas, implementation, through to the uptake of results.

## Method

With several studies now complete, this presentation will focus on strengths of patient-led research. The PaCER method of data collection and analysis engages patients in focus groups to define the scope and research questions (Set), followed by various data collection activities such as focus groups, questionnaires, observation, narrative interviewing (Collect), and a final (Reflect) focus group. Research protocols are negotiated in collaboration with academic researchers, ethics panels, funders and PaCER teams.

## Results

PaCERs have enhanced the research community in tangible ways. Individuals involved in the experience have developed both the self-confidence and competence to be more meaningfully engaged in research outcomes which impact care protocols and the negotiation of healthcare policy. Healthcare professionals have new tools to obtain credible data that are relevant to patients. Based on examples from several recent PaCER studies, this presentation will demonstrate that patients often reveal formerly unknown commentary on how they experience current best practices and entirely refreshing views of optimal treatment, care and outcomes from the patient as consumer perspective.

## Conclusion

The patient engagement research approach will improve the results of patient experience and outcome analysis, in any clinical research area. Adding the active voice, and research minds of patients can enrich the patient engagement process in outcome research.

